# Supplementary material for: Complex‐centric proteome profiling by SEC‐SWATH‐MS
Source: Mol Syst Biol. 2019 Jan 14;15(1):e8438. doi: 10.15252/msb.20188438 (PMC6346213; doi:10.15252/msb.20188438)

mRNA decay complex (UPF1, UPF2, UPF3B, DCP2, XRN1, XRN2, EXOSC2, EXOSC4, EXOSC10, PARN)
Annotated subunits: 10   Subunits with signal: 7
Max. coeluting subunits: 3   Max. completeness: 0.3

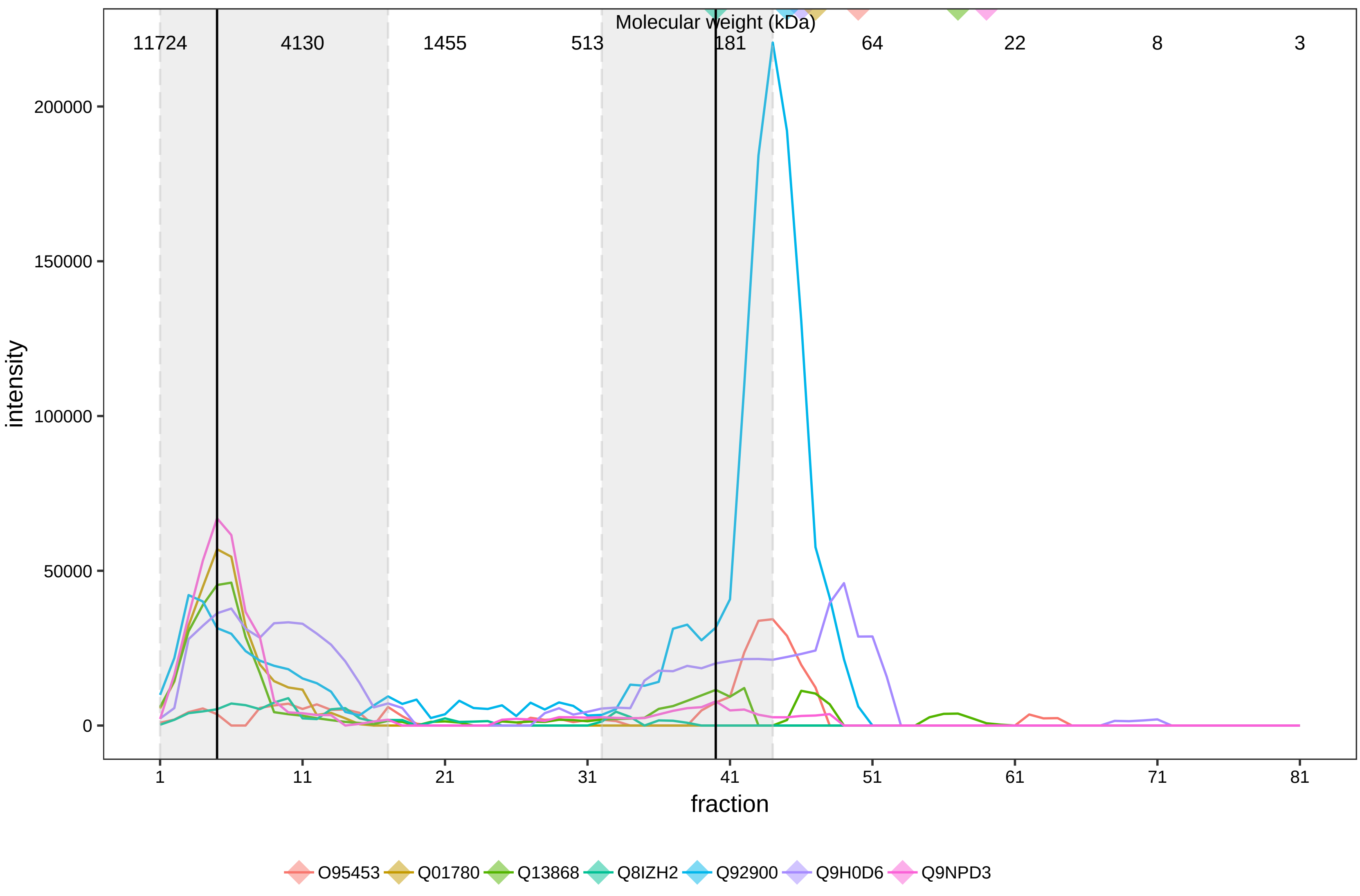

Supplement: Supplementary file 6 — Dataset EV5 [file MSB-15-e8438-s006.zip › feature_plots_corum/822.pdf]
